# False Negative Fecal Occult Blood Test: Prozone Effect

**Published:** 2018-12-27

**Authors:** Pathum Sookaromdee, Viroj Wiwanitkit

**Affiliations:** 1 *TWS Medical Center, Bangkok, Thailand*; 2 *Dr. D. Y. Patil University, Pune, India*

Dear editor, the fecal occult blood test is the presently widely used screening laboratory test for colorectal can- cer. At present, the test is usually based on an immunological diagnostic principle (1, 2). A false positive fecal oc- cult blood is common and widely mentioned in literature. Nevertheless, the false negative is little mentioned in the paper. Here, the authors discuss the issue of the fecal occult blood test’s false negative problem. The case is a consultation on a patient’s laboratory aberration. This patient had hemorrhoids and rectosigmoidoscope showed active bleeding. Nevertheless, the stool occult blood test in this patient always showed a negative result. The physician in charge consulted a clinical pathologist for this problem, and the latter noted that this case is that of a false negative due to the prozone effect. The dilutional preparation of the stool sample is done for confirmation. Before dilutional preparation, the result is negative, and is positive afterwards. In fact, the prozone phenomenon is a common but little mentioned problem in clinical diagnosis. The problem can be seen in serological tests. For the fecal occult blood test, the false negative due to prozone effect is little mentioned in the paper, and the prac- titioner should recognize that there is also a possibility of a false negative in this test. According to the principle behind the fecal occult blood test principle, a hemoglobin concentration above 0.5 mg/mL can cause a prozone effect and a false negative.

## Conflict of Interest

The authors declare that there is no conflict of interest in the publication of this paper.

## References

[1] Quintero E (2009). Chemical or immunological tests for the detection of fecal occult blood in colorectal can- cer screening?. Gastroenterol Hepatol.

[2] Bretagne JF, Manfredi S, Heresbach D (2007). Colorectal cancer mass screening: present and future. Presse Med.

